# Is elective neck dissection justified in cT2N0M0 oral cavity cancer defined according to the AJCC eighth edition staging system?

**DOI:** 10.1002/cam4.6894

**Published:** 2024-01-03

**Authors:** Tsung‐Ming Chen, Shyuang‐Der Terng, Li‐Yu Lee, Shu‐Ru Lee, Shu‐Hang Ng, Chung‐Jan Kang, Jin‐Ching Lin, Chih‐Yen Chien, Chun‐Hung Hua, Cheng Ping Wang, Wen‐Cheng Chen, Yao‐Te Tsai, Chi‐Ying Tsai, Chien‐Yu Lin, Kang‐Hsing Fan, Hung‐Ming Wang, Chia‐Hsun Hsieh, Chih‐Hua Yeh, Chih‐Hung Lin, Chung‐Kan Tsao, Nai‐Ming Cheng, Tuan‐Jen Fang, Shiang‐Fu Huang, Li‐Ang Lee, Ku‐Hao Fang, Yu‐Chien Wang, Wan‐Ni Lin, Li‐Jen Hsin, Tzu‐Chen Yen, Yu‐Wen Wen, Chun‐Ta Liao

**Affiliations:** ^1^ Department of Otolaryngology, Shuang Ho Hospital Taipei Medical University New Taipei City Taiwan, ROC; ^2^ Department of Head and Neck Surgery, Koo Foundation Sun Yat‐Sen Cancer Center Taipei Taiwan, ROC; ^3^ Department of Pathology Chang Gung Memorial Hospital and Chang Gung University Taoyuan Taiwan, ROC; ^4^ Research Service Center for Health Information Chang Gung University Taoyuan Taiwan, ROC; ^5^ Department of Diagnostic Radiology Chang Gung Memorial Hospital and Chang Gung University Taoyuan Taiwan, ROC; ^6^ Department of Otorhinolaryngology, Head and Neck Surgery Chang Gung Memorial Hospital and Chang Gung University Taoyuan Taiwan, ROC; ^7^ Department of Radiation Oncology, Changhua Christian Hospital Changhua Taiwan, ROC; ^8^ Department of Otolaryngology, Chang Gung Memorial Hospital Kaohsiung Medical Center Chang Gung University College of Medicine Taoyuan Taiwan, ROC; ^9^ Department of Otorhinolaryngology China Medical University Hospital Taichung Taiwan, ROC; ^10^ Department of Otolaryngology National Taiwan University Hospital and College of Medicine Taipei Taiwan, ROC; ^11^ Department of Radiation Oncology Chang Gung Memorial Hospital and Chang Gung University Taoyuan Taiwan, ROC; ^12^ Department of Otorhinolaryngology‐Head and Neck Surgery, Chang Gung Memorial Hospital Chiayi Taiwan, ROC; ^13^ Department of Oral and Maxillofacial Surgery, Chang Gung Memorial Hospital Chang Gung University Taoyuan Taiwan, ROC; ^14^ Department of Medical Oncology Chang Gung Memorial Hospital and Chang Gung University Taoyuan Taiwan, ROC; ^15^ Department of Plastic and Reconstructive Surgery Chang Gung Memorial Hospital and Chang Gung University Taoyuan Taiwan, ROC; ^16^ Department of Nuclear Medicine and Molecular Imaging Center Chang Gung Memorial Hospital and Chang Gung University Taoyuan Taiwan, ROC; ^17^ Clinical Informatics and Medical Statistics Research Center Chang Gung University Taoyuan Taiwan, ROC; ^18^ Division of Thoracic Surgery, Chang Gung Memorial Hospital Taoyuan Taiwan, ROC

**Keywords:** cancer registry, clinical outcomes, cT2N0M0, elective neck dissection, occult lymph node metastasis, oral cavity squamous cell carcinoma

## Abstract

**Background:**

The current NCCN guidelines recommend considering elective neck dissection (END) for early‐stage oral cavity squamous cell carcinoma (OCSCC) with a depth of invasion (DOI) exceeding 3 mm. However, this DOI threshold, determined by evaluating the occult lymph node metastatic rate, lacks robust supporting evidence regarding its impact on patient outcomes. In this nationwide study, we sought to explore the specific indications for END in patients diagnosed with OCSCC at stage cT2N0M0, as defined by the AJCC Eighth Edition staging criteria.

**Methods:**

We examined 4723 patients with cT2N0M0 OCSCC, of which 3744 underwent END and 979 were monitored through neck observation (NO).

**Results:**

Patients who underwent END had better 5‐year outcomes compared to those in the NO group. The END group had higher rates of neck control (95% vs. 84%, *p* < 0.0001), disease‐specific survival (DSS; 87% vs. 84%, *p = 0*.0259), and overall survival (OS; 79% vs. 73%, *p* = 0.0002). Multivariable analysis identified NO, DOI ≥5.0 mm, and moderate‐to‐poor tumor differentiation as independent risk factors for 5‐year neck control, DSS, and OS. Based on these prognostic variables, three distinct outcome subgroups were identified within the NO group. These included a low‐risk subgroup (DOI <5 mm plus well‐differentiated tumor), an intermediate‐risk subgroup (DOI ≥5.0 mm or moderately differentiated tumor), and a high‐risk subgroup (poorly differentiated tumor or DOI ≥5.0 mm plus moderately differentiated tumor). Notably, the 5‐year survival outcomes (neck control/DSS/OS) for the low‐risk subgroup within the NO group (97%/95%/85%, *n* = 251) were not inferior to those of the END group (95%/87%/79%).

**Conclusions:**

By implementing risk stratification within the NO group, we found that 26% (251/979) of low‐risk patients achieved outcomes similar to those in the END group. Therefore, when making decisions regarding the implementation of END in patients with cT2N0M0 OCSCC, factors such as DOI and tumor differentiation should be taken into account.

## 1 INTRODUCTION

Elective neck dissection (END) and neck observation (NO) are the two main options for managing neck lymph nodes in early‐stage oral cavity squamous cell carcinoma (OCSCC). According to the National Comprehensive Cancer Network (NCCN) guidelines, END should be strongly considered for cT1 − 2N0M0 tumors exhibiting a depth of invasion (DOI) >3 mm.^1^ However, the evidence supporting this cutoff was based on occult nodal metastatic rates, and the relationship between DOI and survival did not achieve statistical significance.^2^

Patients with a likelihood of more than 20% for occult neck metastases should be offered END.^3^ While the occurrence of occult neck metastases in cT1N0M0 OCSCC is generally less than 15%, this rate significantly rises to over 20% for cT2N0M0 OCSCC.^2^, ^4^ Therefore, even though the justification for END in cT1N0M0 cases may be questionable, it seems prudent to take this approach into account for cT2N0M0 OCSCC. Unfortunately, in their analyses, most published studies on early‐stage OCSCC combined cT1N0 and cT2N0 diseases.^4^, ^5^

In the best‐case scenario, patients undergoing NO would successfully salvage all occult metastases, resulting in a disease‐specific survival (DSS) similar to that of patients receiving END. However, different DSS outcomes related to END were reported in two separate meta‐analyses on early‐stage OCSCC. One the one hand, a meta‐analysis conducted in 2016 on 23 studies and 3244 patients showed that END improved DSS for cT1 − 2 N0 OCSCC.^6^ On the other hand, a 2020 meta‐analysis comprising 41 studies and 5705 patients revealed that there was no significant improvement in DSS with END.^5^ As a result, there is still no consensus in the discussions that surround the use of upfront END in early‐stage OCSCC.

The published literature does not sufficiently address the necessity of END for patients with cT2N0M0 OCSCC, as defined by the AJCC Staging Manual, Eighth Edition.^7^, ^8^ To fill this knowledge gap, a nationwide study was carried out in Taiwan. Although cT2N0M0 patients undergoing END may have significantly different 5‐year outcomes from those receiving NO, our hypothesis was that some patients might be spared END following appropriate risk stratification.

## 2 METHODS

### 2.1 Data sources

We obtained patient data from the Taiwanese Cancer Registry Database (TCRD) “long‐form”, which covers over 99% of Taiwanese patients diagnosed with OCSCC. However, information about salvage therapy for patients experiencing disease relapse is not available. Survival outcome data were collected from the Taiwanese National Health Insurance Research Dataset (TNHIRD). The results of the study are presented in compliance with the Reporting Recommendations for Tumor Marker Prognostic Studies (REMARK) guidelines.^9^, ^10^ The research protocol was approved by the Chang Gung Memorial Hospital's Ethics Committee (reference number: 201801398B0A3), which also granted a waiver for obtaining written informed consent.

### 2.2 Patient selection

**FIGURE 1:**
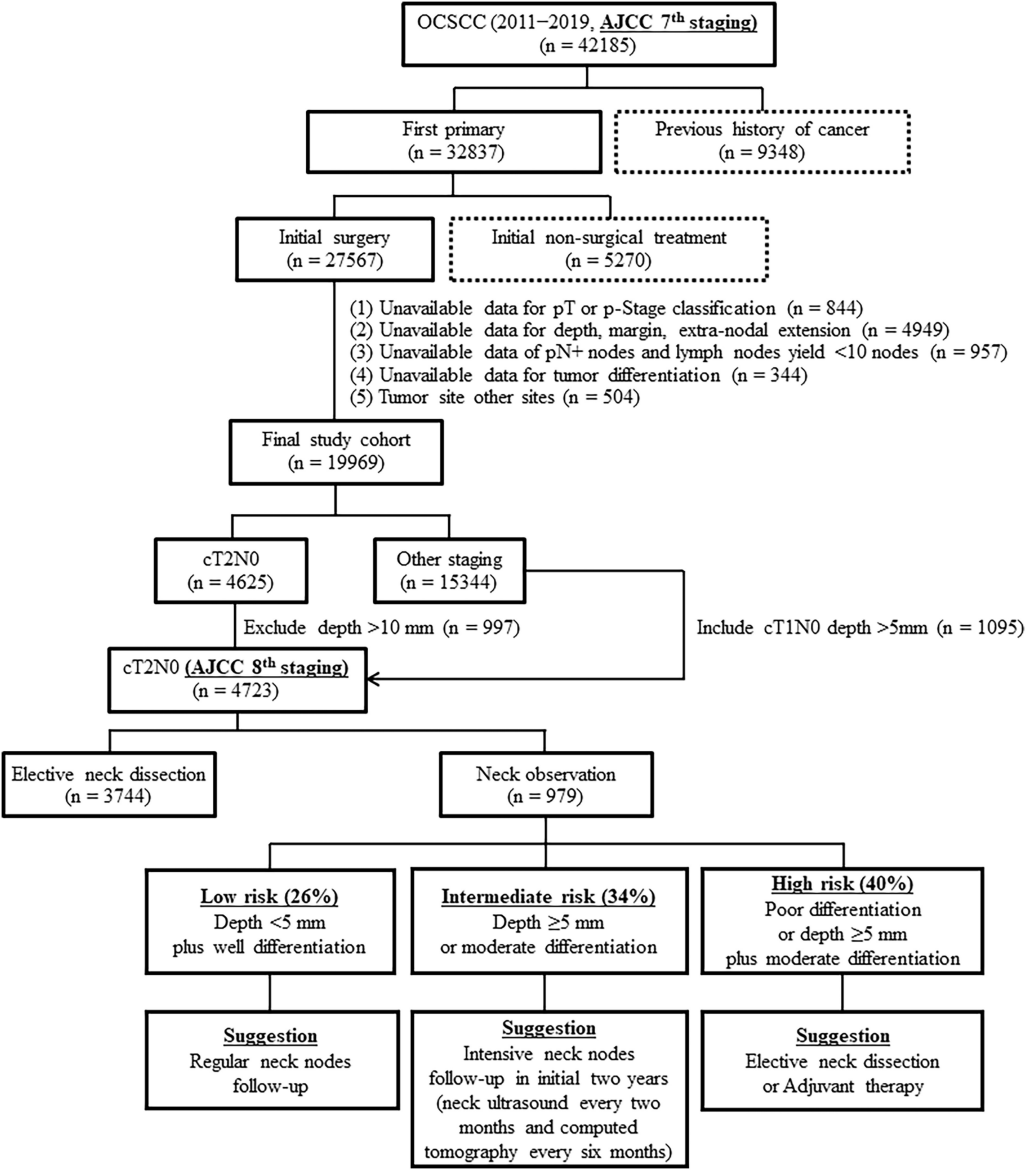
Flow of patients through the study.

We initially considered patients diagnosed with OCSCC from 2011 to 2019, totaling 42,185 individuals. Patient selection was guided by the International Classification of Diseases for Oncology, Third Edition (ICD‐O‐3) codes. A visual representation of the study progression is provided in Figure 1. Initial disease staging was based on the American Joint Committee on Cancer (AJCC) Staging Manual, Seventh Edition (2010). To align with the updated T‐classification guidelines in the AJCC Staging Manual, Eighth Edition (2018), we included an additional 1095 cT1N0 patients (as per the Seventh Edition) with a DOI greater than 5 mm. Concurrently, we excluded 997 cT2N0 patients (as per the Seventh Edition) with a DOI exceeding 10 mm. The final study cohort included 4723 cT2N0M0 OCSCC patients who underwent either END (*n* = 3744) or NO (*n* = 979). The follow‐up period was calculated from the day of surgery until either the patient's death or the end of the study (December 2020).

### 2.3 Data collection

After the study variables were acquired from the TCRD and TNHIRD releases in 2019 and 2020, respectively, data analyses were completed in July 2023. The TCRD adheres to the Standards for Oncology Registry Entry (STORE) manual's guidelines.^11^ Information on OCSCC‐associated morbidity and mortality was obtained from the TNHIRD. Extracted data were then used to compute DSS and overall survival (OS), respectively.

### 2.4 Statistical analysis

Survival curves were plotted with the Kaplan–Meier method and compared using the log‐rank test. Univariable and multivariable Cox proportional hazards regression analyses were applied to assess the associations between the study variables and survival outcomes. A stepwise selection method was employed, incorporating all variables from the univariable analysis into the multivariable model. The results were presented as hazard ratios (HRs) along with their corresponding 95% confidence intervals (CIs). All statistical tests were conducted using a two‐sided approach with a significance level set at 5%.

## 3 RESULTS

### 3.1 Optimal cutoff values for doi

**FIGURE 2:**
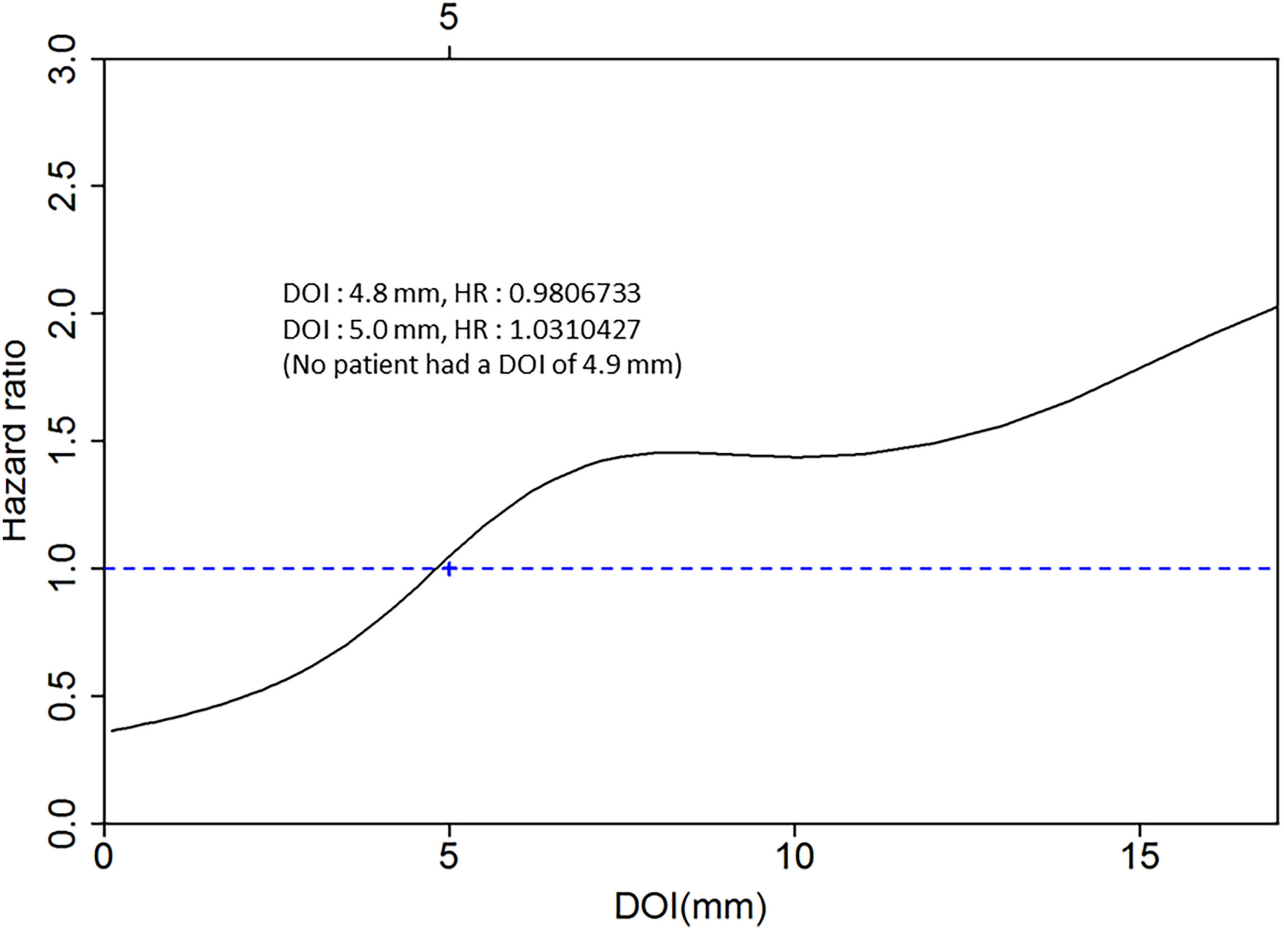
Adjusted hazard ratios of neck control based on the depth of tumor invasion.

Figure 2 illustrates the adjusted HRs for neck control, utilizing a penalized spline method to treat DOI as a continuous variable. This technique enables the evaluation of the non‐linear association between DOI and the log‐transformed HR of neck control. The recursive partitioning analysis determined that a DOI cutoff of 5 mm was optimal in predicting neck control. Notably, when the DOI values reached 5 mm or more, the HRs for neck control exhibited a significant increase.^12^

### 3.2 Patient characteristics

Table 1 presents the general characteristics of patients who underwent either END or NO. The NO group showed a significantly higher prevalence of various factors compared to the END group (all *p* < 0.0001, except for sex). These variables included hard palate and buccal subsites, female sex, age of 65 years or older, well‐differentiated tumors, positive margin and margin status of less than 5 mm, reduced tumor DOI, treatment involving surgery alone, and a weighted charlson comorbidity index (CCI) greater than 1.^13^

**TABLE 1:** General characteristics of patients with oral cavity squamous cell carcinoma who received neck observation or elective neck dissection (*n* = 4723).

Characteristic (*n*, %)	Neck observation (*n* = 979)	Elective neck dissection (*n* = 3744)	*p*
Tumor subsite			<0.0001
Lip (134, 2.8)	34 (3.5)	100 (2.7)	
Tongue (2134, 45.2)	406 (41.5)	1728 (46.2)	
Gum (354, 7.5)	66 (6.7)	288 (7.7)	
Mouth floor (187, 4.0)	18 (1.8)	169 (4.5)	
Hard palate (63, 1.3)	32 (3.3)	31 (0.8)	
Buccal (1641, 34.7)	397 (40.6)	1244 (33.2)	
Retromolar (210, 4.5)	26 (2.6)	184 (4.9)	
Sex			0.0047
Men (4205, 89.0)	847 (86.5)	3358 (89.7)	
Women (518, 11.0)	132 (13.5)	386 (10.3)	
Age (years)			<0.0001
<65 (3660, 77.5)	659 (67.3)	3001 (80.2)	
≥65 (1063, 22.5)	320 (32.7)	743 (19.8)	
Tumor differentiation			<0.0001
Well (1479, 31.3)	447 (45.7)	1032 (27.6)	
Moderate (2935, 62.1)	485 (49.5)	2450 (65.4)	
Poor (309, 6.6)	47 (4.8)	262 (7.0)	
Margin status (mm)			<0.0001
Positive (169, 3.6)	76 (7.8)	93 (2.5)	
<5 (2322, 49.2)	621 (63.4)	1701 (45.4)	
≥5 (2232, 47.2)	282 (28.8)	1950 (52.1)	
Depth of invasion (mm)			<0.0001
Range 0.1–17.0, mean: 5.9, median: 6			
Mean ± standard deviation	5.29 ± 3.08	6.10 ± 2.87	
Treatment modality			<0.0001
S alone (3580, 75.8)	829 (84.7)	2751 (73.5)	
S plus adjuvant therapy^a^ (1143, 24.2)	150 (15.3)	993 (26.5)	
Weighted Charlson comorbidity index			
Mean ± standard deviation	1.06 ± 1.44	0.77 ± 1.13	<0.0001
0–1 (3790, 80.2)	724 (74.0)	3066 (81.9)	
>1 (933, 19.8)	255 (26.0)	678 (18.1)	
Lymph nodes			
Nodal yield number		Range: 10 to 90, mean: 29.5, median: 27	
Number of metastatic lymph nodes		Range: 0–22, mean: 0.3, median: 0	

Abbreviations: CT, chemotherapy; RT, radiotherapy; S, surgery.

^a^ S plus CT or S plus RT or S plus CT plus RT.

### 3.3 Rates of occult metastases according to the ajcc staging manual, seventh, and eighth editions

After evaluating 4625 patients diagnosed with cT2N0M0 OCSCC, staged according to the AJCC Staging Manual, Seventh Edition, we found that out of the 3875 patients who underwent ND, 748 (19.3%) displayed occult metastases. In contrast, when analyzing 4723 patients with cT2N0M0 OCSCC according to the AJCC Staging Manual, Eighth Edition, we observed that among the 3744 patients who underwent ND, 598 (16.0%) had occult metastases.

### 3.4 Five‐year survival rates

**FIGURE 3:**
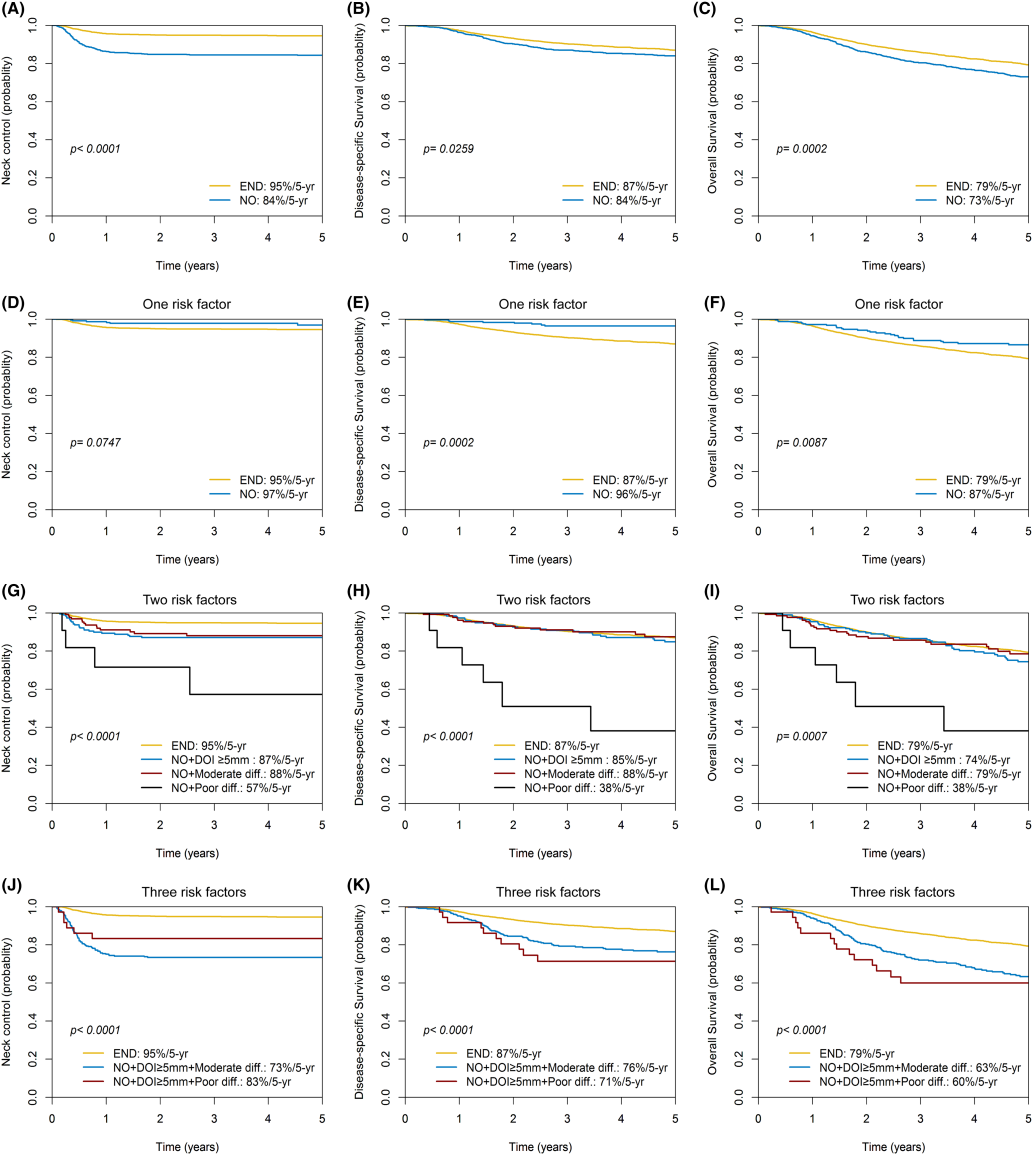
Kaplan–Meier plots of 5‐year neck control, disease‐specific survival, and overall survival for patients who had undergone elective neck dissection versus neck observation prior to risk stratification (A − C), elective neck dissection versus neck observation and one independent risk factor (D − F), elective neck dissection versus neck observation and two independent risk factors (G − I), as well as elective neck dissection versus neck observation and three independent risk factors (J − L).

In the entire study cohort, the rates for 5‐year neck control, DSS, and OS were 92%, 86%, and 78% respectively. A comparison between patients in the END and NO groups revealed significant differences in these outcomes. For those who underwent END, the 5‐year neck control, DSS, and OS rates were 95%, 87%, and 79%, respectively. In contrast, the rates for those who received NO were 84% (*p* < 0.0001), 84% (*p* = 0.0259), and 73% (*p* = 0.0002), respectively (Figure 3A–C).

### 3.5 Univariable and multivariable cox regression analysis

Table 2 presents the results of univariable and multivariable analyses. After adjustment for potential confounders in multivariable analysis, several factors were independently associated with a decreased 5‐year neck control rate. These included NO, a DOI of 5 mm or more, moderately‐to‐poorly differentiated tumors, and treatment with surgery alone. A less favorable 5‐year DSS was independently associated to NO, a DOI of 5 mm or more, moderately‐to‐poorly differentiated tumors, a positive margin, treatment with surgery plus adjuvant therapy, and a CCI greater than 1. Finally, a decreased 5‐year OS was found to be independently linked to NO, a DOI of 5 mm or more, an age of 65 years or older, moderately‐to‐poorly differentiated tumors, a positive margin and margin of less than 5 mm, treatment with surgery plus adjuvant therapy, and a CCI greater than 1 (Table 2).

**TABLE 2:** Univariable and multivariable analyses of risk factors for 5‐year neck control, disease‐specific survival, and overall survival in patients with cT2N0M0 oral cavity squamous cell carcinoma (*n* = 4723).

Characteristic	Neck control				Disease‐specific survival				Overall survival			
	Univariable analysis		Stepwise multivariable analysis		Univariable analysis		Stepwise multivariable analysis		Univariable analysis		Stepwise multivariable analysis	
	HR (95% CI)	*p*	HR (95% CI)	*p*	HR (95% CI)	*p*	HR (95% CI)	*p*	HR (95% CI)	*p*	HR (95% CI)	*p*
Neck treatment												
Neck observation	3.20 (2.57–3.98)	<0.0001	3.80 (3.04–4.74)	<0.0001	1.23 (1.03–1.47)	0.0262	1.40 (1.15–1.69)	0.0006	1.30 (1.13–1.50)	0.0002	1.29 (1.11–1.50)	0.0007
Elective neck dissection	1		1		1		1		1		1	
Depth of invasion (mm)												
< 5.0	1		1		1		1		1		1	
≥ 5.0	2.35 (1.75–3.16)	<0.0001	2.46 (1.82–3.32)	<0.0001	1.93 (1.59–2.35)	<0.0001	1.64 (1.34–2.01)	<0.0001	1.58 (1.37–1.82)	<0.0001	1.42 (1.23–1.65)	<0.0001
Tumor subsite												
Lip	1		–		1		–		1		–	
Tongue	1.61 (0.80–3.27)	0.1843	–	ns	0.82 (0.54–1.25)	0.3555	–	ns	0.76 (0.54–1.06)	0.1056	–	ns
Gum	0.61 (0.26–1.46)	0.2687	–	ns	0.59 (0.35–0.98)	0.0424	–	ns	0.68 (0.46–1.01)	0.0552	–	ns
Mouth floor	0.85 (0.33–2.15)	0.7252	–	ns	0.63 (0.35–1.15)	0.1305	–	ns	0.87 (0.56–1.34)	0.5263	–	ns
Hard palate	0.71 (0.19–2.68)	0.6128	–	ns	0.56 (0.24–1.29)	0.1725	–	ns	0.85 (0.48–1.51)	0.5725	–	ns
Buccal	0.62 (0.30–1.29)	0.1972	–	ns	0.61 (0.39–0.93)	0.0226	–	ns	0.62 (0.44–0.87)	0.0056	–	ns
Retromolar	0.50 (0.18–1.38)	0.1807	–	ns	0.75 (0.44–1.28)	0.2902	–	ns	0.69 (0.45–1.06)	0.0891	–	ns
Sex												
Men	0.68 (0.50–0.92)	0.0137	–	ns	0.87 (0.68–1.10)	0.2365	–	ns	1.01 (0.83–1.22)	0.9565	–	ns
Women	1		–		1		–		1		1	
Age (years)												
< 65	1		–		1		–		1		1	
≥ 65	1.19 (0.93–1.54)	0.1710	–	ns	1.30 (1.09–1.56)	0.0036	–	ns	1.71 (1.49–1.95)	<0.0001	1.56 (1.36–1.78)	<0.0001
Tumor differentiation												
Well	1		1		1		1		1		1	
Moderate	2.11 (1.58–2.81)	<0.0001	2.35 (1.75–3.15)	<0.0001	1.65 (1.36–1.99)	<0.0001	1.48 (1.22–1.81)	<0.0001	1.43 (1.24–1.65)	<0.0001	1.37 (1.18–1.58)	<0.0001
Poor	2.65 (1.71–4.10)	<0.0001	3.34 (2.13–5.21)	<0.0001	2.94 (2.22–3.89)	<0.0001	2.25 (1.68–3.01)	<0.0001	2.37 (1.89–2.96)	<0.0001	1.97 (1.56–2.49)	<0.0001
Margin status (mm)												
Positive	0.76 (0.39–1.50)	0.4322			2.29 (1.65–3.18)	<0.0001	1.69 (1.20–2.38)	0.0025	2.10 (1.60–2.75)	<0.0001	1.65 (1.25–2.18)	0.0005
< 5	1.01 (0.81–1.25)	0.9569	–	ns	1.25 (1.07–1.47)	0.0059	1.17 (0.995–1.38)	0.0577	1.23 (1.09–1.39)	0.0012	1.17 (1.03–1.33)	0.0147
≥ 5	1		–		1		1		1		1	
Treatment modality												
Surgery alone	1		1		1		1		1		1	
Surgery plus adjuvant therapy^a^	0.57 (0.43–0.77)	0.0003	0.52 (0.38–0.70)	<0.0001	2.10 (1.79–2.46)	<0.0001	1.77 (1.49–2.09)	<0.0001	1.67 (1.47–1.90)	<0.0001	1.48 (1.29–1.69)	<0.0001
Weighted Charlson comorbidity index												
0–1	1		–		1		1		1		1	
>1	1.20 (0.92–1.55)	0.1803	–	ns	1.35 (1.13–1.63)	0.0012	1.33 (1.11–1.60)	0.0022	1.74 (1.52–1.99)	<0.0001	1.58 (1.37–1.81)	<0.0001

Abbreviations: CI, confidence interval; HR, hazard ratio; ns, not significant.

^a^ Surgery plus chemotherapy or surgery plus radiotherapy or surgery plus chemotherapy plus radiotherapy.

### 3.6 Occult metastatic rates according to various patient subgroups

The rates of occult metastasis exhibited distinct patterns within different patient subgroups. Specifically, patients with a DOI <5 mm had a rate of 9.0% (97/1076), whereas those with a DOI ≥5 mm experienced a higher rate of 18.8% (501/2668). Furthermore, the rates varied based on tumor differentiation, with well‐differentiated tumors having a rate of 8.0% (82/1032), moderately differentiated tumors at 17.5% (429/2450), and poorly differentiated tumors exhibiting the highest rate at 33.2% (87/262).

### 3.7 Comparison of five‐year survival rates in patients stratified according to independent risk factors (RFs)

Multivariable analysis identified three independent RFs for all of the three outcomes of interest, that is, NO, a DOI of 5 mm or more, and moderately‐to‐poorly differentiated tumors. For a DOI of less than 5 mm, the 5‐year neck control, DSS, and OS rates were 96%, 92%, and 85%, respectively. Conversely, for a DOI of 5 mm or more, these rates were 91%, 84%, and 75%, respectively (all *p* < 0.0001). On analyzing tumor differentiation, the 5‐year rates were 96%, 91%, and 89% for neck control, 91%, 85%, and 73% for DSS, and 84%, 77%, and 64% for OS for well, moderately, and poorly differentiated tumors, respectively (all *p* < 0.0001). We used the 5‐year outcomes of END as reference benchmarks (neck control: 95%, DSS: 87%, OS: 79%) to identify specific subgroups of patients who underwent NO and yet achieved comparable rates. The outcomes for NO subgroups with one, two, and three RFs are detailed in Figure 3 and Table 3. Patients who underwent NO with a DOI of less than 5 mm and well‐differentiated tumors had 5‐year neck control, DSS, and OS rates of 97%, 96%, and 87%, respectively (*n* = 251). These rates were not lower than those of patients who received END (95%, 87%, and 79%; Figure 3D–F). The 5‐year DSS and OS rates for patients who underwent NO with a DOI of 5 mm or more and well‐differentiated tumors (85% and 74%, *n* = 196) or NO with moderately differentiated tumors and a DOI of less than 5 mm (88% and 79%, *n* = 132) were comparable to those of patients who received END (87% and 79%; Figure 3H,I). However, the 5‐year neck control, DSS, and OS rates for NO with poorly differentiated tumors and a DOI of less than 5 mm (57%, 38%, and 38%, *n* = 11; Figure 3G–I), NO with a DOI of 5 mm or more and moderately differentiated tumors (73%, 76%, and 63%, *n* = 353), and NO with a DOI of 5 mm or more and poorly differentiated tumors (83%, 71%, and 60%, *n* = 36) were significantly lower than those of patients who received END (Figure 3J–L).

**TABLE 3:** Five‐year neck control, disease‐specific survival, and overall survival in the elective neck dissection group versus distinct neck observation subgroups.

Subgroup/(number)	Neck control		Disease‐specific survival		Overall survival	
	HR (95% CI)	*p*	HR (95% CI)	*p*	HR (95% CI)	*p*
Elective neck dissection (*n* = 3744)	Reference (95%/5‐year)		Reference (87%/5‐year)		Reference (79%/5‐year)	
Neck observation						
+ DOI <5 mm + well differentiated tumor (*n* = 251)	0.49 (0.22–1.10)	0.0816	0.35 (0.20–0.62)	0.0003	0.64 (0.46–1.90)	0.0093
Neck observation						
+ DOI ≥5 mm + well differentiated tumor (*n* = 196)	2.56 (1.67–3.92)	<0.0001	1.10 (0.76–1.59)	0.6158	1.17 (0.89–1.55)	0.2697
Neck observation						
+ moderately differentiated tumor + DOI <5 mm (*n* = 132)	2.22 (1.29–3.83)	0.0040	0.998 (0.62–1.62)	0.9940	1.13 (0.79–1.62)	0.4935
Neck observation						
+ poorly differentiated tumor + DOI <5 mm (*n* = 11)	9.30 (3.45–25.05)	<0.0001	7.06 (3.15–15.80)	<0.0001	4.40 (1.97–9.83)	0.0003
Neck observation						
+ DOI ≥5 mm + moderately differentiated tumor (*n* = 353)	6.04 (4.69–7.78)	<0.0001	1.88 (1.48–2.38)	<0.0001	1.84 (1.52–2.22)	<0.0001
Neck observation						
+ DOI ≥5 mm + poorly differentiated tumor (*n* = 36)	3.65 (1.62–8.23)	0.0018	2.46 (1.31–4.59)	0.0049	2.46 (1.47–4.09)	0.0006

Abbreviations: CI, confidence interval; DOI, depth of invasion; HR, hazard ratio.

## 4 DISCUSSION

In this study, we found that patients with cT2N0M0 OCSCC who underwent END had better neck control compared to those who received NO (95% versus 84%, respectively; *p* < 0.0001). However, the differences in DSS between the two groups were only slightly significant (87% vs. 84%, respectively; *p* = 0.0259). The most likely reason for this marginal significance is the successful salvage of cervical metastases in the NO group. In the published literature, the reported salvage rates for neck metastatic lesions vary considerably from less than 50%^2^ to 75%–100%.^14^, ^15^, ^16^, ^17^ Unfortunately, we are unable to reach a firm conclusion on this issue because salvage therapy data are not included in the TCRD.

The SEER study of cT2N0M0 OCSCC staged in accordance with the AJCC Seventh Edition found, in contrast to the current investigation, that there was a roughly 10% difference in the 5‐year DSS rate between those who received NO and those who underwent END. As a result, in order to manage cT2N0M0 OCSCC, the SEER investigators recommended using END.^7^ It is generally expected that, the differences in DSS between patients undergoing END and those receiving NO are anticipated to decrease in proportion to the prevalence of occult cervical metastases. In this study, we observed that Taiwanese patients with cT2N0M0 OCSCC had a significantly lower prevalence of occult neck metastases (16.0%) than most other patients in the literature, where rates typically surpass 20%.^4^ In addition, the rate of occult metastasis observed in our investigation (16.0%) fell below the 20% cutoff point that Weiss et al.^3^ recommended as the threshold at which END would be appropriate. This could lead to the suggestion that, for Taiwanese patients with cT2N0M0 OCSCC staged in accordance with the AJCC Staging Manual, Eight Edition, NO might be adequate.

Crucially, our results also showed that the highest rates of occult metastasis were found in patients with poorly differentiated tumors (33.2%) and a DOI of 5 mm or more (18.8%). Consequently, in order to guarantee the best possible clinical outcomes, END should view as essential for these patients. In contrast, patients who had well‐differentiated tumors (8.0%) and a DOI of less than 5 mm (9.5%) exhibited the lowest rates of occult metastasis, thereby making them suitable candidates for NO. These findings are supported by the positive outcomes observed in this subgroup, with their 5‐year rates for neck control, DSS, and OS being comparable to those who underwent END (97%/96%/87% versus 95%/87%/79%, respectively; Figure 3D–F and Table 3). In light of the lack of significant prognostic differences for this specific subgroup, NO in conjunction with regular neck node follow‐up would be a reasonable approach (Figure 1, low‐risk subgroup).

Within the NO group, patients with a moderately differentiated tumor or a DOI of 5 mm or more had neck control rates that were significantly lower than those of patients who underwent END (87%–88% vs. 95%, respectively; Figure 3G and Table 3). Nevertheless, the 5‐year DSS and OS rates for patients with a DOI of 5 mm or more (85%/74%) and moderate differentiation (88%/79%) were consistent with the outcomes of patients who underwent END (87%/79%; Figure 3 H‐I and Table 3). Cervical metastases should have a high chance of being salvaged in these two NO subgroups. Therefore, it is essential to closely monitor the neck lymph nodes for the first 2 years after primary tumor excision. This objective can be met with CT scans performed every 6 months and neck ultrasonography exams every 2 months. Additionally, fine needle cytology can be used as needed to assist in the early detection of lymph node metastases that are salvageable (Figure 1, intermediate‐risk subgroup). In comparison to cases that received END, the 5‐year neck control, DSS, and OS rates were significantly lower for patients undergoing NO with a poorly differentiated tumor (regardless of DOI <5 mm versus ≥5 mm) or NO with a DOI ≥5 mm and a moderately differentiated tumor. There is a lower chance of successfully rescuing cervical metastases and a higher likelihood of hidden metastatic spread in these three particular subgroups. Therefore, END should be carried out whenever possible following primary tumor removal. If the patient's overall health status precludes END, adjuvant therapy should be taken into account as an alternative (Figure 1, high‐risk subgroup).

Nonetheless, it is crucial to consider that a trustworthy evaluation of tumor differentiation and DOI requires permanent section pathology for tumor excision. As such, tumor removal and END ought to be carried out in two distinct stages. While END is anticipated to result in better outcomes and may be relatively simpler to perform in clinically negative necks, there is a considerable risk of morbidity associated with this procedure. Additionally, up to 80% of patients may not benefit from this approach.^18^ To solve this conundrum, better risk stratification is therefore required.

In this study, independent RFs for 5‐year DSS and OS were found to include a positive margin/margin status of less than 5 mm and a CCI >1. These variables, however, had no appreciable prognostic impact on neck control. As a result, we did not include these two parameters in the neck treatment outcome analysis. Tumor margins have a limited influence on neck control, but they are a major prognostic factor for local control.^19^ An additional noteworthy finding is that the NO group exhibited a greater number of patients with a lower tumor DOI, a positive margin, and a margin status of less than 5 mm. These findings suggest that patients who received NO had less invasive tumors that were less aggressively treated. However, to guarantee favorable clinical outcomes, surgical margins should always be considered. Notably, fewer patients in the NO group received adjuvant therapy, despite the fact that there were more cases with positive margins and margin status <5 mm (Table 1). Making recommendations about postoperative adjuvant therapy for patients in the NO group is difficult due to the lack of information regarding occult lymph node metastasis in this patient group. This highlights how important it is to pinpoint high‐risk patients who could benefit from adjuvant therapy. Notably, adjuvant therapy has been identified as an independent adverse risk factor for 5‐year DSS and OS, as opposed to surgery alone. This illustrates how this patient group's disease is generally more advanced (e.g., pN+ versus pN0). Since this was not the primary goal of the study, no additional subgroup analysis was carried out on patients who received adjuvant therapy or not and who had varying margin status.

The NCCN guidelines state that a DOI greater than 3 mm should be taken into consideration when deciding whether to perform an upfront END for cT1 − 2N0M0 OCSCC.^1^, ^2^ Notably, the substantial rise in the cumulative rate of occult nodal metastases—from ≥3 mm (5.6%) to ≥4 mm (16.9—was taken into consideration when choosing the level 1 evidence supporting this cutoff for DOI.^2^ However, further analysis showed that the interaction between disease‐free survival and DOI, considered as a continuous variable, did not reach the statistical significance threshold (Supplementary Table S 3a, HR: 1.29 [0.91–1.85], *p* = 0.15).^2^ In the current investigation, the rate of occult nodal metastases increased from 9% for a DOI <5 mm to 18.8% for a DOI ≥5 mm. Moreover, we showed that neck control, DSS, and OS were affected by a 5 mm DOI cutoff. Here, we also found that the optimal cutoff value for DOI (5 mm) exceeded that recommended by the NCCN guidelines. This could be explained by the utilization of different AJCC staging manuals (Seventh versus Eighth Edition) and different patient stages (cT1 − 2N0M0 versus cT2N0M0) in the analysis. Interestingly, poor tumor differentiation is not listed as a separate adverse prognostic variable for OCSCC in the NCCN guidelines. Tumor differentiation, however, has been suggested as a crucial factor to take into account when evaluating neck control, clinical outcomes, and the occurrence of occult neck metastases.^20^

We identified three independent RFs (NO, DOI ≥5 mm, and moderately‐to‐poorly differentiated tumors) for 5‐year neck control, DSS, and OS. Based on the prognostic scoring system we developed, DOI and tumor differentiation should be considered when deciding whether to perform END in patients with cT2N0M0 disease. Based on these criteria, we found that 26% (251/979) of the study participants could have avoided END without compromising oncological outcomes; 34% (328/979) of patients could have had favorable 5‐year DSS and OS with rigorous neck node follow‐up; and 40% (400/979) of patients needed adjuvant therapy or END in order to achieve more satisfactory outcomes (Figure 1 and Figure 3).

There are limitations to this study. First, we were unable to analyze the impact of salvage therapy on clinical outcomes. Second, it was not possible to evaluate the prognostic significance of perineural invasion, lymphatic invasion, and vascular invasion, which are known to be independent adverse RFs for neck control, DSS, and OS in patients with OCSCC.^20^ Given that data on lympho‐vascular invasion and perineural invasion have only been gathered since 2018, more research is required to ascertain their prognostic value.

In conclusion, this study suggests that tumor differentiation and DOI should be taken into consideration when deciding whether to perform an END in patients with cT2N0M0 OCSCC. Interestingly, favorable outcomes comparable to those in the END group were attained by at least 26.0% of patients in the NO group.

## AUTHOR CONTRIBUTIONS

**Tsung‐Ming Chen:** Conceptualization (lead); data curation (lead); formal analysis (lead); investigation (lead); methodology (lead); project administration (lead); resources (lead); software (lead); validation (lead); visualization (lead); writing – original draft (lead); writing – review and editing (lead). **Shyuang‐Der Terng:** Conceptualization (lead); data curation (lead); formal analysis (lead); investigation (lead); methodology (lead); project administration (lead); resources (lead); software (lead); supervision (lead); validation (lead); visualization (lead); writing – review and editing (lead). **Li‐Yu Lee:** Conceptualization (equal); data curation (equal); formal analysis (equal); methodology (equal); resources (equal); software (equal); supervision (equal); validation (equal); writing – original draft (equal). **Shu‐Ru Lee:** Conceptualization (equal); data curation (equal); formal analysis (equal); methodology (equal); resources (equal); software (equal); supervision (equal); validation (equal); writing – original draft (equal). **Shu‐Hang Ng:** Conceptualization (equal); data curation (equal); formal analysis (equal); methodology (equal); resources (equal); software (equal); supervision (equal); validation (equal); writing – original draft (equal). **Chung‐Jan Kang:** Conceptualization (equal); data curation (equal); formal analysis (equal); methodology (equal); resources (equal); software (equal); supervision (equal); validation (equal); writing – original draft (equal). **Jin‐Ching Lin:** Conceptualization (equal); data curation (equal); formal analysis (equal); methodology (equal); resources (equal); software (equal); supervision (equal); validation (equal); writing – original draft (equal). **Chih‐Yen Chien:** Conceptualization (equal); data curation (equal); formal analysis (equal); methodology (equal); resources (equal); software (equal); supervision (equal); validation (equal); writing – original draft (equal). **Chun‐Hung Hua:** Conceptualization (equal); data curation (equal); formal analysis (equal); methodology (equal); resources (equal); software (equal); supervision (equal); validation (equal); writing – original draft (equal). **Cheng Ping Wang:** Conceptualization (equal); data curation (equal); formal analysis (equal); methodology (equal); resources (equal); software (equal); supervision (equal); validation (equal); writing – original draft (equal). **Wen‐Cheng Chen:** Conceptualization (equal); data curation (equal); formal analysis (equal); methodology (equal); resources (equal); software (equal); supervision (equal); validation (equal); writing – original draft (equal). **Yao‐Te Tsai:** Conceptualization (equal); data curation (equal); formal analysis (equal); methodology (equal); resources (equal); software (equal); supervision (equal); validation (equal); writing – original draft (equal). **Chi‐Ying Tsai:** Conceptualization (equal); data curation (equal); formal analysis (equal); methodology (equal); resources (equal); software (equal); supervision (equal); validation (equal); writing – original draft (equal). **Chien‐Yu Lin:** Conceptualization (equal); data curation (equal); formal analysis (equal); methodology (equal); resources (equal); software (equal); supervision (equal); validation (equal); writing – original draft (equal). **Kang‐Hsing Fan:** Conceptualization (equal); data curation (equal); formal analysis (equal); methodology (equal); resources (equal); software (equal); supervision (equal); validation (equal); writing – original draft (equal). **Hung‐Ming Wang:** Conceptualization (equal); data curation (equal); formal analysis (equal); methodology (equal); resources (equal); software (equal); supervision (equal); validation (equal); writing – original draft (equal). **Jason Chia‐Hsun Hsieh:** Conceptualization (equal); data curation (equal); formal analysis (equal); methodology (equal); resources (equal); software (equal); supervision (equal); validation (equal); writing – original draft (equal). **Chih‐Hua Yeh:** Conceptualization (equal); data curation (equal); formal analysis (equal); methodology (equal); resources (equal); software (equal); supervision (equal); validation (equal); writing – original draft (equal). **Chih‐Hung Lin:** Conceptualization (equal); data curation (equal); formal analysis (equal); methodology (equal); resources (equal); software (equal); supervision (equal); validation (equal); writing – original draft (equal). **Chung‐Kan Tsao:** Conceptualization (equal); data curation (equal); formal analysis (equal); methodology (equal); resources (equal); software (equal); supervision (equal); validation (equal); writing – original draft (equal). **Nai‐Ming Cheng:** Conceptualization (equal); data curation (equal); formal analysis (equal); methodology (equal); resources (equal); software (equal); supervision (equal); validation (equal); writing – original draft (equal). **Tuan‐Jen Fang:** Conceptualization (equal); data curation (equal); formal analysis (equal); methodology (equal); resources (equal); software (equal); supervision (equal); validation (equal); writing – original draft (equal). **Shiang‐Fu Huang:** Conceptualization (equal); data curation (equal); formal analysis (equal); methodology (equal); resources (equal); software (equal); supervision (equal); validation (equal); writing – original draft (equal). **Li‐Ang Lee:** Conceptualization (equal); data curation (equal); formal analysis (equal); methodology (equal); resources (equal); software (equal); supervision (equal); validation (equal); writing – original draft (equal). **Ku‐Hao Fang:** Conceptualization (equal); data curation (equal); formal analysis (equal); methodology (equal); resources (equal); software (equal); supervision (equal); validation (equal); writing – original draft (equal). **Yu‐Chien Wang:** Conceptualization (equal); data curation (equal); formal analysis (equal); methodology (equal); resources (equal); software (equal); supervision (equal); validation (equal); writing – original draft (equal). **Wan‐Ni Lin:** Conceptualization (equal); data curation (equal); formal analysis (equal); methodology (equal); resources (equal); software (equal); supervision (equal); validation (equal); writing – original draft (equal). **Li‐Jen Hsin:** Conceptualization (equal); data curation (equal); formal analysis (equal); methodology (equal); resources (equal); software (equal); supervision (equal); validation (equal); writing – original draft (equal). **Tzu‐Chen Yen:** Conceptualization (equal); data curation (equal); formal analysis (equal); methodology (equal); resources (equal); software (equal); supervision (equal); validation (equal); writing – original draft (equal). **Yu‐Wen Wen:** Conceptualization (lead); data curation (lead); formal analysis (lead); funding acquisition (lead); investigation (lead); methodology (lead); project administration (lead); resources (lead); software (lead); supervision (lead); validation (lead); visualization (lead); writing – original draft (lead). **Chun‐Ta Liao:** Conceptualization (lead); data curation (lead); formal analysis (lead); investigation (lead); methodology (lead); project administration (lead); resources (lead); software (lead); supervision (lead); validation (lead); visualization (lead); writing – original draft (lead); writing – review and editing (lead).

## CONFLICT OF INTEREST STATEMENT

The authors declare no conflicts of interest that could potentially affect the presentation or interpretation of the research findings in this study.

## FUNDING INFORMATION

This study was financially supported by grants (CMRPD1H0521 and BMRPC55) from the Chang Gung Medical Research Program.

## ETHICS STATEMENT

This study was approved by the Chang Gung Memorial Hospital's Ethics Committee (reference number: 201801398B0A3).

## CONSENT

The Chang Gung Memorial Hospital's Ethics Committee deemed it unnecessary to obtain written informed consent from participants due to the retrospective nature of the study.

## Data Availability

The study's data accessibility is restricted by third parties. According to the Taiwanese Ministry of Health and Welfare's Health and Welfare Data Center (http://dep.mohw.gov.tw/DOS/), data sharing is prohibited by law due to the “Personal Information Protection Act”. Nonetheless, a license to use the data for research purposes was given to the authors. If formal permission from the Taiwanese Ministry of Health and Welfare is obtained, the corresponding author will make the datasets created and/or analyzed during this study available upon reasonable request.
